# Calcineurin: The Achilles’ heel of fungal pathogens

**DOI:** 10.1371/journal.ppat.1011445

**Published:** 2023-07-06

**Authors:** Vikas Yadav, Joseph Heitman

**Affiliations:** Department of Molecular Genetics and Microbiology, Duke University Medical Center, Durham, North Carolina, United States of America; University of Melbourne, AUSTRALIA

## What is calcineurin?

**Fig 1:**
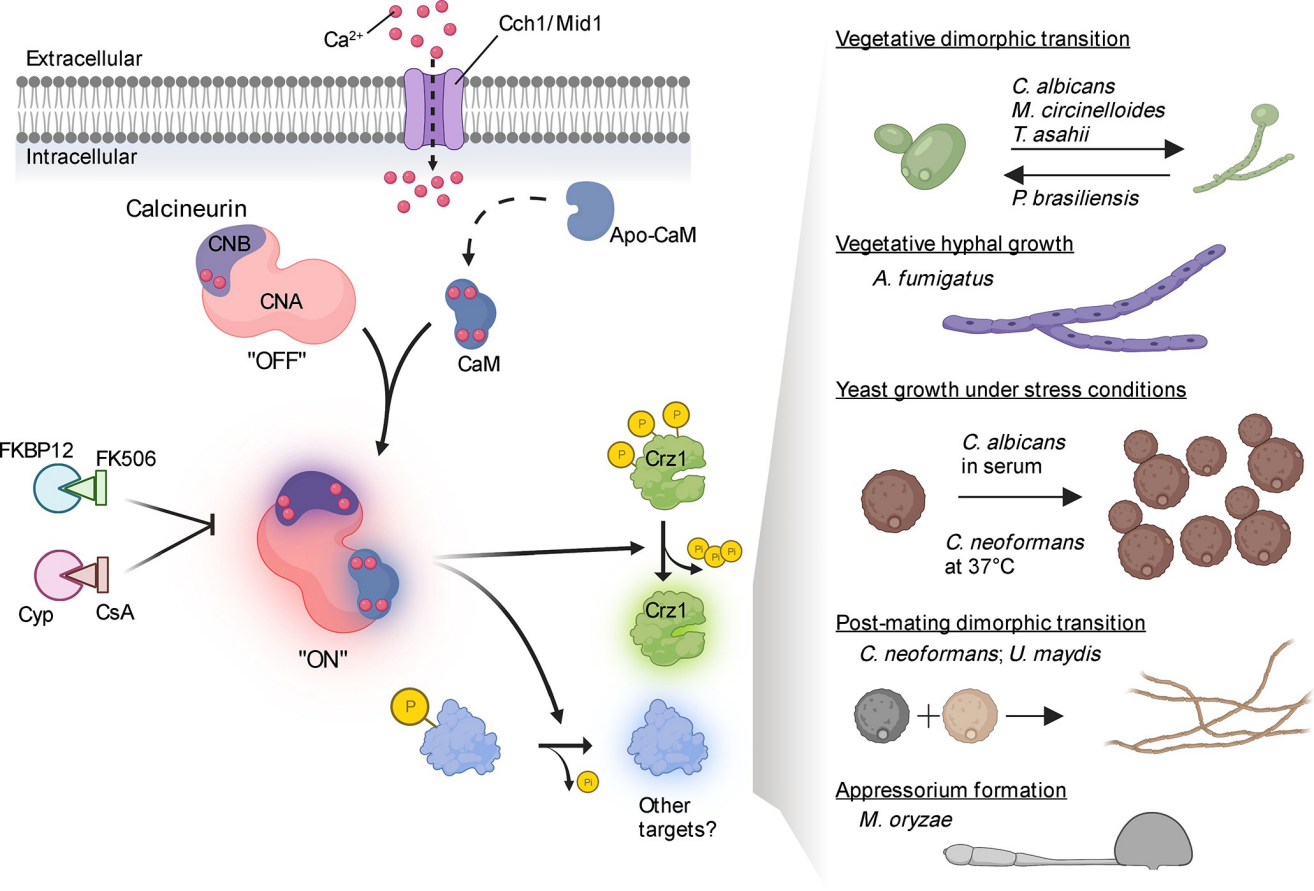
**Calcineurin signaling in fungal pathogens.** Calcineurin signaling is initiated upon calcium influx and binding of calcium-calmodulin (CaM) to the calcineurin complex (CNA and CNB). The binding of CaM releases an autoinhibitory domain that occupies the active site in the catalytic subunit CNA converting calcineurin from an inactive “OFF” configuration to a fully active “ON” state. Upon activation, calcineurin dephosphorylates its substrates, which include a conserved transcription factor, Crz1, and several others that remain unidentified in most fungal species. Through these targets, calcineurin governs stress responses, morphological transitions, and virulence in fungal pathogens of humans and plants. As described in the right-side panel, calcineurin directs yeast–hyphal dimorphic transitions in some fungi, whereas it is required for vegetative yeast or hyphal growth in other fungal species. In some cases, calcineurin orchestrates the development of specialized structures, such as formation of appressoria in *M*. *oryzae*, that are required for infection and virulence. Calcineurin activity can be inhibited by FK506 and cyclosporine (CsA), both of which are immunosuppressive drugs when bound to FKBP12 or cyclophilin A (Cyp). The figure was created with BioRender.com.

Calcineurin is a highly conserved serine-threonine specific protein phosphatase and was named based on its discovery as a calcium- and calmodulin-binding protein that is highly abundant in the central nervous system (calci+neurin) [1]. Calcineurin is a heterodimer comprised of a catalytic A subunit (CNA) and a regulatory B subunit (CNB) [2,3]. In the basal state, CNB is bound by 2 Ca^2+^ ions and calcineurin is kept inactive through an autoinhibitory domain (AID) that loops into the active site of CNA [2] (Fig 1). Upon calcium influx, CNB binds to 2 additional Ca^2+^ ions causing structural changes in the CNA-CNB heterodimer. Finally, the heterodimer is associated with another calcium-binding protein, calmodulin, which itself is primed via structural changes through binding of 4 Ca^2+^ ions. Calmodulin binding triggers the release of the AID from the catalytic site of the enzyme rendering calcineurin fully active. Once activated, calcineurin binds to its substrates by docking onto 2 short-linear motifs, PxIxIT and LxVP, and dephosphorylates substrates to govern their subcellular localization and functions [3].

## How important is calcineurin for fungal pathogens?

Calcineurin plays key roles in fungi in response to calcium influx and is the only known calcium-responsive phosphatase. Calcineurin has been studied in several fungal pathogens of both humans and plants and plays crucial roles in stress adaptation and pathogenicity (Fig 1 and Table 1). In the human fungal pathogen, *Candida albicans*, calcineurin is required for survival in serum and plays an important role during hyphal growth required to cause infections [4–6]. Additionally, loss of function or inhibition of calcineurin also results in enhanced susceptibility to antifungal drugs in widespread clinical use such as fluconazole. Calcineurin plays a crucial role in the hyphal growth and virulence of *Aspergillus fumigatus*, a filamentous fungal pathogen [7]. The catalytic subunit, CNA, localizes to the active sites of hyphal growth and septum formation, playing an important role in growth and septation in *A*. *fumigatus* [8]. Inhibiting calcineurin activity also results in hypersensitivity to other cell wall inhibitors in this fungus suggesting a major role for calcineurin in proper cell wall synthesis and repair [9].

**Table 1:** Calcineurin functions in fungal pathogens.

Fungal pathogen	Calcineurin functions	References
Human pathogens		
*Aspergillus fumigatus*	Hyphal growth, cell wall integrity, **septation, cation homeostasis**, antifungal drug resistance, and virulence	[7–9,22]
*Candida albicans*	Hyphal growth, ER stress response, cell wall integrity, azole tolerance, **growth in serum,** and **virulence**	[4–6,20,34,37]
*Candida glabrata*	Thermotolerance, ER stress response, cell wall integrity, **azole tolerance**, **growth in serum**, and **virulence**	[38,39]
*Cryptococcus neoformans*	**Thermotolerance**, cell wall integrity, antifungal drug tolerance, **postmating dimorphic transition from yeast to hyphae**, and **virulence**	[10,11,21,24]
*Mucor circinelloides*	Vegetative dimorphic transition from yeast to hyphae, hyphal growth, antifungal drug resistance, and virulence	[14,40]
*Paracoccidioides brasiliensis*	Vegetative dimorphic transition from hyphae to yeast, yeast and hyphal growth, and calcium homeostasis	[13]
*Talaromyces marneffei*	Hyphal growth, conidiation and conidia germination, cell wall integrity, osmotic stress response, survival in macrophages, and virulence	[16]
*Trichosporon asahii*	Thermotolerance, cell wall integrity, ER stress response, hyphal formation, and virulence	[15]
Plant pathogens		
*Botrytis cinerea*	Conidiation, cation homeostasis, cell wall integrity, and virulence	[41]
*Magnaporthe oryzae*	Mycelial growth, conidiation, **appressorium formation**, and virulence	[17,23,42]
*Ustilago maydis*	Postmating dimorphic transition from yeast to hyphae, and virulence	[18]
*Ustilago hordei*	Thermotolerance, cell wall integrity, cation homeostasis, pH stress, and virulence	[43]

Functions listed in **bold** indicate roles that are known to be either completely or partially independent of Crz1.

In *Cryptococcus neoformans*, mutation or inhibition of calcineurin renders cells inviable at 37°C, and avirulent in mice [10]. In addition, calcineurin is also required for hyphal growth in *C*. *neoformans*, which is essential for completing the sexual cycle and producing infectious spores [11]. Microscopy studies demonstrated that calcineurin re-localizes to septation sites as well as processing-bodies (P-bodies) and stress granules during 37°C heat stress [12]. Calcineurin has also been found to play essential roles in virulence in several other human fungal pathogens including *Mucor circinelloides*, *Paracoccidioides brasiliensis*, *Talaromyces marneffei*, and *Trichosporon asahii* [13–16].

*Magnaporthe oryzae* is a serious threat to global food security and is responsible for approximately a 30% loss in rice productivity each year. *M*. *oryzae* infects plant leaves through specialized structures called appressoria. Calcineurin is required for the formation of appressoria and therefore, it is essential for pathogenesis [17]. Calcineurin also plays a critical role in the pathogenicity of *Ustilago maydis*, a plant fungal pathogen that infects corn [18]. *U*. *maydis* undergoes a postmating morphological transition from yeast to hyphal growth that is essential for virulence. Mutation of calcineurin results in the production of multi-budded yeast cells that fail to form hyphae and are thus unable to cause infections. Overall, calcineurin directs stress responses and morphological changes in each of these pathogens and thus serves as a globally conserved virulence factor.

## What are calcineurin’s substrates?

One of the most conserved calcineurin targets in fungi is Crz1, a zinc-finger transcription factor, which is a homolog of NFAT in humans [19]. Upon dephosphorylation by activated calcineurin, Crz1 translocates into the nucleus and regulates the transcription of target genes involved in stress responses. While Crz1 is known to perform roles in the calcineurin pathway in several fungal species, its activity fails to account for all of calcineurin’s functions. For example, Crz1 in *C*. *albicans* plays roles in pH sensitivity, hyphal growth, and drug tolerance but is not required for virulence, unlike calcineurin [20]. Similarly, Crz1 deletion does not lead to the same level of thermal sensitivity as deletion or inhibition of calcineurin in *C*. *neoformans* [21]. In addition, Crz1 is not required for hyphal growth and sporulation in this species, in contrast to calcineurin. While both calcineurin and the Crz1 homolog CrzA play an important role in the virulence of *A*. *fumigatus*, CrzA deletion does not phenocopy the severe hyphal growth defect of the calcineurin mutant, and accordingly, calcineurin plays a more prominent role in cell wall biosynthesis compared to the more restricted role of Crz1 [22]. Interestingly, the Crz1 homolog in *M*. *oryzae* is not required for appressorium formation [23], but Crz1 mutation still reduces rice infection rates. Crz1 in *U*. *maydis* remains to be identified. Overall, these studies demonstrate that Crz1 is a conserved and key downstream effector of calcineurin in most fungal pathogens, but also emphasizes that additional substrates of calcineurin must also play critical roles in stress responses.

Surprisingly, aside from Crz1, most calcineurin targets in pathogenic fungi remain understudied and poorly characterized, except for some potential candidates identified in *C*. *neoformans*. A phosphoproteome study in this fungus identified several calcineurin targets, in addition to Crz1 [24]. Many of these substrates localize to P-bodies/stress granules and are predicted to play important roles in RNA processing. Additionally, substrates involved in septation and vesicle trafficking were also identified, in accord with previous localization studies [12,25]. Analysis of calcineurin substrates revealed that both Crz1 and P-body/stress granule targets contribute to thermal stress adaptation and virulence but their mutation confers only intermediate phenotypes in comparison to wild-type and calcineurin mutants [24]. While Crz1 controls the expression of several cell wall biosynthesis genes in a calcineurin-dependent manner, thus regulating cell wall integrity, the roles of calcineurin in P-bodies/stress granules are not well understood but may involve RNA metabolism. Interestingly, combined mutation of Crz1 and P-body targets does not recapitulate the phenotypes of calcineurin deletion, suggesting that additional substrates may be important for calcineurin functions [24]. A recent study revealed that Crz1 also localizes to stress granules and septa upon heat stress, similar to calcineurin [26]. Interestingly, the study found that Crz1 localizes to stress granules prior to its translocation to the nucleus hinting at the possibility that calcineurin might be dephosphorylating Crz1 at stress granules.

## Can calcineurin serve as a drug target?

Calcineurin, due to its diverse and critical roles, is an attractive antifungal drug target candidate. Due to its stimulatory role in human T-cells, it is an established target of 2 immunosuppressive drugs, FK506 and cyclosporine (CsA), both of which inhibit calcineurin as drug–protein complexes after binding to their respective immunophilin targets, FKBP12, and cyclophilin A [2]. Both FK506 and CsA also exhibit broad antifungal activity highlighting the potential of calcineurin as an antifungal drug target [27]. However, the conservation of calcineurin signaling between humans and fungi requires alternative approaches to identify aspects for fungal-specific targeting. Recently, structure-guided synthetic analogs of FK506 have been developed that demonstrate greater relative specificity for fungal calcineurin than its human counterpart [28–30]. Strengthening this proof-of-concept, these analogs retain antifungal activity with reduced immunosuppressive activity signifying that this approach could yield drug candidate leads. Such an approach of structure-guided synthesis of inhibitors can also be applied to target downstream components of calcineurin, such as Crz1. Another approach could be to identify unique structural and/or sequence features that differ between fungi and the host for drug targeting. Interestingly, a filamentous fungal-specific phosphorylated serine proline-rich (SPRR) domain was identified in *A*. *fumigatus* CNA homolog, which is important for regular hyphal growth, revealing unique protein sequence features that could be exploited for drug development [31]. These findings have revealed a hidden potential of calcineurin inhibitors and suggest more extensive research involving structure-guided drug design is warranted.

## What remains to be learned about calcineurin in pathogenic fungi?

Fungal infections pose a significant challenge to both global human health and food security [32,33]. This threat is further magnified by the increasing population of immunocompromised people and increasing resistance of fungal pathogens to existing treatment options. This scenario suggests that more research is needed to identify novel fungal-specific drug targets that could lead to the development of fungicidal drugs. Studying and characterizing calcineurin in detail in key fungal pathogens has the potential to identify fungal-specific drug targets within this signaling network. However, at present, there is a substantial gap between our knowledge of calcineurin’s roles in fungi and how these are performed at a molecular level. Most research has focused on the functions of calcineurin in cell wall integrity pathways and studying the contributions of Crz1 as a downstream effector. While both of these factors are important and necessary components of the signaling network, they are not sufficient to fully explain the role of calcineurin in fungal pathogenesis. Therefore, more focused and mechanistic approaches are required to critically dissect the roles of calcineurin and its substrates to provide important breakthroughs.

Future studies should delineate a more complete repertoire of calcineurin substrates in fungi. This may result in the identification of calcineurin effectors that are required for virulence but might be specific to a fungal species. In addition, characterizing these effectors in multiple species could also reveal a set of substrates or functions that are specific to fungi and absent from humans. For example, calcineurin interactions at septa appear to be conserved across fungi and thus reflect interactions with fungal-specific targets [8,12]. In addition, such studies may also reveal the basis of the requirement for calcineurin in specific host niches. For example, the requirement for calcineurin in *C*. *albicans* virulence in mice depends on the mode of infection, where it is essential for systemic infection, but not for pulmonary or vaginal infection [4,5,34]. Such studies could help delineate specific host factors that contribute to the establishment of infections revealing interesting infectious disease biology involving calcineurin signaling. This might include delineating roles of calcineurin effectors in different host niches allowing the development of effector-targeting drugs that might be condition specific.

Another exciting aspect of calcineurin signaling that is understudied is the documentation of kinases that phosphorylate calcineurin substrates and thereby regulate their function. For example, the kinase responsible for phosphorylating Crz1 and required for its nuclear export in fungal pathogens remains uncharacterized. Interestingly, 2 separate kinases, Hrr25, and protein kinase A, are known to phosphorylate Crz1 in *S*. *cerevisiae* suggesting that there may be more than 1 Crz1-regulating kinase in other fungi as well [19,35]. Calmodulin is known to also activate several kinases in response to calcium signaling [36] and some of these kinases may phosphorylate calcineurin’s substrates. Recognizing and studying these negative regulators of calcineurin signaling will be important and may lead to the identification of core calcineurin effectors that play essential roles in fungal pathogenesis.

Calcineurin plays a fundamental role in virtually every fungal pathogen studied to date and yet research into this pathway can be accelerated. Going forward, further research into calcineurin signaling will be necessary to fully exploit the therapeutic potential of this essential virulence network. Targeting calcium–calcineurin signaling has the potential to significantly influence not only human health and global food resources but also biodiversity because of its significance in a broad range of fungal pathogens. As a true Achilles’ heel, calcineurin signaling can be aimed at and targeted to counter the emerging threats of fungal diseases.
